# Adherence to WHO guidelines on severe pneumonia management in children and its impact on outcome: an observational study at Jinka General Hospital in Ethiopia

**DOI:** 10.3389/fpubh.2023.1189684

**Published:** 2023-07-27

**Authors:** Adriano La Vecchia, Bereket Gebremedhin Teklie, Dagmawi Awoke Mulu, Kusse Koirita Toitole, Francesca Montalbetti, Carlo Agostoni, Tesfayesus Tefera Hessebo, Ademe Tsegaye, Andrea Pietravalle, Fabio Manenti, Francesca Tognon, Luigi Pisani, Eleni Hagos

**Affiliations:** ^1^Department of Clinical Sciences and Community Health (DISCCO), University of Milan, Milan, Italy; ^2^Jinka General Hospital, Jinka, South Omo, Ethiopia; ^3^Doctors with Africa CUAMM, Jinka, South Omo, Ethiopia; ^4^Fondazione IRCCS Ca' Granda Ospedale Maggiore Policlinico, Pediatric Area, Milan, Italy; ^5^Doctors With Africa CUAMM, Addis Ababa, Ethiopia; ^6^Operational Research Unit, Doctors With Africa CUAMM, Padua, Italy

**Keywords:** Africa, children, Ethiopia, guidelines, pneumonia, treatment, WHO

## Abstract

**Introduction:**

Poor adherence to guidelines during empirical antibiotic prescription in low-income countries could increase antimicrobial resistance without improving outcomes. Revised World Health Organization (WHO) guidelines published in 2014 on childhood (2–59 months) pneumonia re-defined the classification of severe pneumonia and changed the first-line treatment. The adherence to WHO guidelines in southern Ethiopia at the hospital level is unknown. We sought to determine the adherence to WHO guidelines on severe pneumonia first-line treatment in children in an Ethiopian referral hospital and assess the impact of non-adherence on patient outcomes.

**Methods:**

An observational study was conducted on all children (2–59 months) clinically diagnosed with severe pneumonia and admitted to the Pediatric Ward of Jinka Hospital from 1 June 2021 to 31 May 2022. Exclusion criteria included a known HIV infection, ongoing antibiotic treatment before the event not related to acute pneumonia, or any other severe bacterial infection, confirmed or suspected. Adherence to guidelines was defined as first-line treatment with ampicillin or benzylpenicillin and gentamicin at the recommended dose. We compared the patients treated adherently vs. non-adherently. For categorical variables, the chi-square or Fisher's exact test was used, while for continuous variables, the Mann–Whitney U-test was used. Multivariate logistic regression was used to evaluate the association between adherence and demographic and clinical characteristics.

**Results:**

During the observational period, 266 patients were registered as having severe pneumonia with an age between 2 and 59 months. After excluding 114 patients due to missing charts or other exclusion criteria, a total of 152 patients were included in the analysis. Of these, 78 (51%) were girls with a median age of 10 months (IQR 7–14). Overall, 75 (49%) patients received therapy according to the WHO guidelines. Compared to patients treated adherently to the guidelines, patients not treated adherently had similar outcomes [median length of stay of 3 (IQR 3–5) and 4 (IQR 3–6) days], median duration of oxygen therapy of 2 (IQR 1–3) for both the groups, and self-discharge rates of 5% and 6.5%, respectively).

**Conclusion:**

Adherence to the revised WHO guideline was limited and not associated with outcomes. Efforts should focus on reducing the gap between theory and practice.

## 1. Introduction

Sub-Saharan Africa has the world's highest rate of under-5 mortality, estimated in 2019 at 75.8 deaths per 1,000 live births, or one in every 13 children dying before reaching the age of 5 years (1). Among children aged 1–59 months, lower respiratory infections are the leading cause of death (2). Lower respiratory infections may present with different clinical symptoms such as cough, fatigue, obstruction, and respiratory distress with or without fever (3). Ethiopia is one of the five countries where nearly half of all under-5 deaths occurred in 2019 (1), and pneumonia causes 18% of all deaths in this age group (4).

The revised World Health Organization (WHO) guidelines on childhood pneumonia (2 months−5 years) published in 2014 re-defined the classification of severe pneumonia and changed the first-line treatment (5). Once diagnosed, pneumonia is classified as severe if one or more of the following symptoms are present: inability to drink, persistent vomiting, convulsions, lethargy or unconsciousness, stridor in a calm child, or severe malnutrition. The first-line recommended treatment for severe pneumonia is the parenteral combination of ampicillin or benzylpenicillin and gentamicin 7.5 mg/kg once a day for at least 5 days. Ceftriaxone should be used only as a second-line treatment in case of failure of the first-line treatment (5).

The WHO defined antimicrobial resistance (AMR) as one of the biggest threats to public health of the 21st-first century, and antibiotic misuse is one of the major drivers of AMR (6, 7). Antibiotics are the most frequently prescribed drugs worldwide (8), with a high rate of inappropriate prescriptions reported by various authors (9–11). In Ethiopia, most antibiotic prescriptions are empirically made, and the prescribing pattern is non-compliant with the WHO standards (12, 13).

Two previous studies, one in a northern region and another in a southern region of Ethiopia, found poor adherence to the Integrated Community Case Management of Newborn and Child Illness strategy in primary care settings (health posts and health centers), but the management of pneumonia at the hospital level was not investigated (14, 15). Little is known about adherence to guidelines on child pneumonia treatment at the hospital level in southern Ethiopia. Our study aimed to fill this knowledge gap, and the objectives were as follows: (1) to determine adherence to the WHO guidelines for severe pneumonia first-line treatment in under-5 children in a referral hospital in southern Ethiopia and (2) to compare the impact on patient outcomes of adherent and non-adherent empirical antibiotic treatments.

The findings will benefit local practitioners and health actors, as well as serve as a baseline study for any further studies on this topic in the area.

## 2. Materials and methods

### 2.1. Design, setting, and patients

We performed a retrospective observational study at Jinka General Hospital, a referral hospital located in Jinka Town, South Omo Zone, Southern Nations, Nationalities, and Peoples Region.

The South Omo Zone is an area of ~2.3 million hectares in southern Ethiopia, bordering Kenya and South Sudan. Based on the 2022 Ethiopian Statistical Service projections (16), the South Omo Zone has a population of more than 800,000 inhabitants living in a traditional agro-pastoral system of subsistence. The population pyramid has a large base with a high prevalence of children under the age of five (17). The Pediatric Ward at Jinka Hospital consists of 19 beds and admits an average of 850 patients annually.

The study examined clinical records of children admitted with pneumonia from 1 June 2021 to 31 May 2022.

In Jinka General Hospital, clinical information is recorded on paper. Patients to be included were identified from the admission register of the Pediatric Ward. Data were then extracted from the patient chart with the help of the chart room employee. Three trained physicians analyzed patient charts, collecting demographic, clinical, and therapeutic data in a standardized case report form.

The inclusion criteria were a pediatric ward admission of a patient aged 2–59 months with a clinical diagnosis of severe pneumonia during the observational period. The diagnosis was considered starting from the registry and was subsequently confirmed by the data from the medical record.

Exclusion criteria were (1) incomplete information (missing medical record), (2) a known HIV infection, (3) an ongoing antibiotic treatment before the admission not correlated with acute pneumonia, and (4) other concomitant severe bacterial infections confirmed or suspected (e.g., sepsis and meningitis).

We evaluated the presence of concurrent chronic diseases, such as cardiovascular disease, pulmonary disease, neurologic disease, and metabolic disease, which were reported as comorbidities.

During data collection, the authors had access to information that could identify individual participants. The Jinka University Ethical Committee approved the study (reference number JKU/RCE/ERC/055/15), which included a waiver of informed consent because of the retrospective nature of the investigation.

### 2.2. Study endpoints

We evaluated the adherence to the WHO guidelines for first-line treatment with ampicillin or benzylpenicillin and gentamicin at the recommended dose (5).

Three different indicators were considered for the outcome evaluation: oxygen therapy days, hospitalization days, and death in the hospital.

### 2.3. Data analysis

Descriptive statistics were performed. Continuous data were presented as median and interquartile range and categorical data as numbers and percentages (18, 19). We compared the patients' outcome indicators between patients treated in adherence to the WHO guidelines and other patients. We also evaluated self-discharges as possible confounders. The chi-square test or Fisher's exact test was used for categorical variables, and the Mann–Whitney U-test was used for continuous ones. We used a non-parametric test after verifying that the continuous variables were not normally distributed using the Shapiro–Wilk normality test. We used multivariate logistic regression to evaluate the association between adherence to the guideline (as the dependent variable) and the independent variables such as sex, age, referral status, and patient severity (defined as the presence of central cyanosis/oxygen saturation <90%, severe respiratory distress, or lethargy). We found no significant sources of bias in the study's design. We performed analyses based only on the available data.

A *p*-value of <0.05 was considered statistically significant. Statistical analysis was performed using R software (version 3.6.3 for Windows).

## 3. Results

### 3.1. Patient cohort

During the observational period, 266 patients aged between 2 and 59 months were recorded in the pediatric ward registry as having severe pneumonia at admission. Considering the number of patients, they represent ~30% of all admissions.

**Figure 1:**
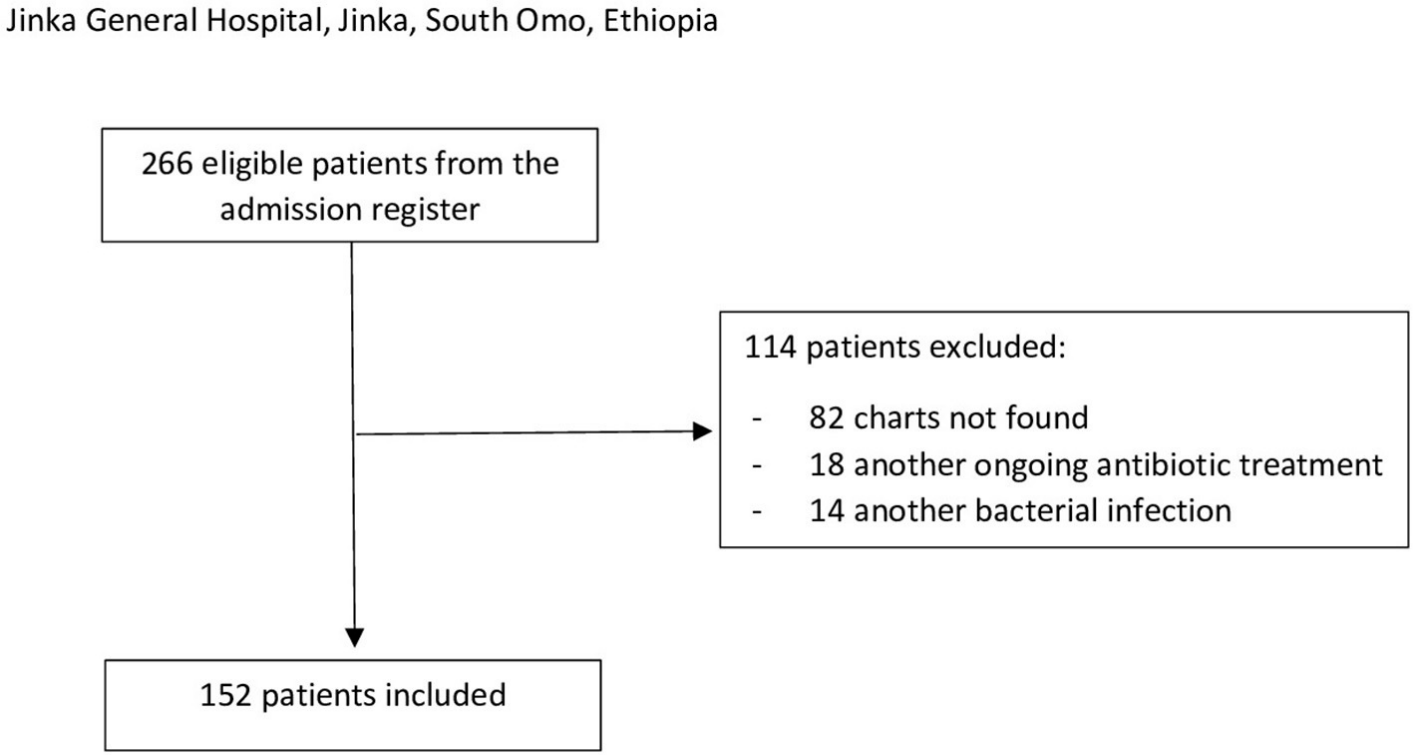
Inclusion/exclusion flowchart. The inclusion criteria were a pediatric ward admission of a patient aged 2–59 months with a clinical diagnosis of severe pneumonia during the observational period (1 June 2021 to 31 May 2022). Exclusion criteria were (1) incomplete information (missing medical record), (2) a known HIV infection, (3) an ongoing antibiotic treatment before the admission not correlated with acute pneumonia, and (4) other concomitants severe bacterial infections confirmed or suspected (e.g., sepsis and meningitis).

Among them, 114 patients were excluded: 82 patients' charts were not found, 18 patients were on another ongoing antibiotic therapy, and 14 had other suspected or confirmed severe bacterial infections (Figure 1). None of the patients was registered as HIV positive.

A total of 152 patients were included in the study, 78 (51%) girls and 74 (49%) boys, with a median age of 10 months (IQR 7–14). The included patients had no missing data. Five (3%) patients were referred from other health centers, and seven (5%) patients had comorbidities. Table 1 shows the demographic and clinical characteristics of the sample.

**Table 1:** Sample characteristics divided by sex and total sample.

	**F (*n =* 78)**	**M (*n =* 74)**	**TOT (*n* =152)**
**Baseline characteristics**			
Age, median [IQR]	9 [6–15.5]	10.5 [7–14]	10 [7–14]
Comorbidity	4 (5)	3 (4)	7 (5)
Referred	2 (3)	3 (4)	5 (3)
**Clinical characteristics**, ***n*** **(%)**			
Central cyanosis or oxygen saturation <90%	45 (58)	33 (45)	78 (51)
Severe respiratory distress	64 (82)	46 (62)	110 (72)
Inability to drink	1 (1)	1 (1)	2 (1)
Persistent vomiting	9 (11.5)	11 (15)	20 (13)
Convulsions	0	0	0
Lethargy or unconsciousness	16 (20.5)	14 (19)	30 (20)
Stridor in a calm child	1 (1)	1 (1)	2 (1)
Severe malnutrition	1 (1)	1 (1)	2 (1)
No WHO severe defining symptoms	7 (9)	12 (16)	19 (12.5)
**Outcomes**			
Oxygen days, median [IQR]	2 [1–3]	2 [0–3]	2 [1–3]
Hospitalization days, median [IQR]	3 [3–6]	3.5 [3–5]	3 [3–6]
Recovery	77 (99)	66 (89)	143 (94)
Self-discharge	1 (1)	8 (11)	9 (6)

Data are presented as median [interquartile range] or as frequency (percentage).

At least one of the WHO's severe defining symptoms was present in 133 (87.5%) patients. The most prevalent symptom was severe respiratory distress reported in 110 (72%) patients, followed by central cyanosis or oxygen saturation <90% in 78 (51%) patients, lethargy or unconsciousness in 30 (20%) patients, persistent vomiting in 20 (13%) patients, inability to drink in 2 (1%) patients, stridor in a calm child in 2 (1%) patients, and severe malnutrition in 2 (1%) patients. No patients presented with convulsion, and 19 (12.5%) of the patients did not report any severe defining symptom.

### 3.2. Adherence to WHO guidelines

Overall, 75 (49%) patients received therapy according to the WHO guidelines. The most commonly used antibiotic treatment was a combination of benzylpenicillin plus gentamicin in 72 (47%) patients, followed by ceftriaxone plus gentamicin in 42 (28%), and ceftriaxone alone in 20 (13%) patients. Table 2 shows the therapeutic schemes. Using a multivariate logistic regression model, we did not find any significant association between adherence to the WHO guidelines and sex (OR 0.8, CI 0.4–1.6), age (OR 1, CI 0.9–1), referral status (OR 1.4, CI 0.2–11.2), central cyanosis or saturation <90% (OR 1.1, CI 0.6–2.1), severe respiratory distress (OR 0.9, CI 0.4–2.1), and lethargy or unconsciousness (OR 0.8, CI 0.4–1.9).

**Table 2:** First-line antibiotic treatments used.

**Antibiotic treatment**	***n* (%)**
Ampicillin + gentamicin	3 (2)
Benzylpenicillin+ gentamicin	72 (47)
Benzylpenicillin alone	3 (2)
Benzylpenicillin+ gentamicin+ azithromycin	3 (2)
Ceftriaxone alone	20 (13)
Ceftriaxone + gentamicin	42 (28)
Ceftriaxone + gentamicin + azithromycin	1 (1)
Ceftriaxone + azithromycin	7 (5)
Ceftriaxone + metronidazole	1 (1)

Data are presented as frequency (percentage). Ampicillin + gentamicin and benzylpenicillin+ gentamicin are the schemes suggested by the revised WHO guidelines.

### 3.3. Association with outcomes

No deaths were registered among the sample cohort, 143 (94%) patients recovered and 9 (6%) patients were self-discharged. The median duration of oxygen therapy was 2 (IQR 1–3) days, and the median length of stay was 3 (IQR 3–6) days.

We compared patients who received therapy as adherent to the WHO guidelines vs. those who did not. Demographics, clinical characteristics, and outcomes were similar between the two groups (Table 3). We found no significant difference in oxygen days, hospitalization days, or self-discharges between patients treated according to the revised WHO guidelines and those who were not.

**Table 3:** Baseline, clinical characteristics, and outcomes by adherence to the revised WHO guidelines.

	**Non-adherent therapy (*n* = 77)**	**Adherent therapy (*n* = 75)**	***p*-value**
**Baseline characteristics**			
Female	38 (49)	40 (53)	0.6
Male	39 (51)	35 (47)	
Comorbidity	4 (5)	3 (4)	1
Primary admission	75 (97)	72 (96)	0.6
Referred	2 (3)	3 (4)	
Age in months, median [IQR]	10 [7–14]	9 [6–13.5]	0.1
**Clinical characteristics**, ***n*** **(%)**			
Central cyanosis or oxygen saturation <90%	39 (51)	39 (52)	0.9
Severe respiratory distress	56 (73)	54 (72)	0.9
Inability to drink	1 (1)	1 (1)	1
Persistent vomiting	14 (18)	6 (8)	0.09
Lethargy or unconsciousness	16 (21)	14 (19)	0.7
Stridor in a calm child	1 (1)	1 (1)	1
Severe malnutrition	1 (1)	1 (1)	1
No WHO severe defining symptoms	7 (9)	12 (16)	0.2
**Outcomes**			
Oxygen days, median [IQR]	2 [1–3]	2 [1–3]	0.1
Hospitalization days, median [IQR]	4 [3–6]	3 [3–5]	0.5
Recovery	72 (93.5)	71 (95)	1
Self-discharge	5 (6.5)	4 (5)	1

Data are presented as median [interquartile range] or as frequency (percentage).

Regarding the other risk factors, we reported a significant association between male sex and self-discharge: eight (10%) male patients and one (1%) female patient self-discharged (OR 9.3, CI 1.6–175.4). When we evaluated the association between outcomes and adherence to guidelines adjusting for sex as possible confounders, we did not find a correlation (OR 0.9, CI 0.2–3.5).

## 4. Discussion

To the best of our knowledge, this is the first study to evaluate adherence to the WHO-revised guidelines on severe pneumonia in children at the hospital level in southern Ethiopia. We found that only half of the patients received an antibiotic treatment adherent to the guideline. Other studies evaluated adherence to the previous WHO guidelines in sub-Saharan Africa. A Sudanese study demonstrated poor adherence (18.8%) to severe pneumonia treatment during 2009–2010 in an urban children's referral hospital in Khartum (20). Similarly, a Kenyan study found a low level of adherence (27.7%) to the treatment of pneumonia in children in the Pediatric Department of Garissa Provincial General Hospital in Kenya (21). Our results do not allow us to understand the reason for the low adherence to guidelines, but we can exclude the lack of antibiotic supply since benzylpenicillin, ampicillin, and gentamicin were available continuously during the observational period.

We found no difference in hospitalization days, oxygen therapy, or outcomes when children were treated following the guidelines or using alternative antibiotics, most commonly ceftriaxone which is considered second-line treatment. These results are consistent with the Sudanese study (20). Our data show that outcomes of patients treated accordingly to the WHO guideline are not inferior to those treated with different antibiotic schemes and that physicians did not prescribe broader spectrum antibiotics to more severe patients.

We had no case fatalities in our sample cohort, although nine (6%) patients self-discharged. With a few exceptions, such as malnourished patients, patients older than 1 month in Ethiopia must pay for any treatment they receive, and sometimes the family cannot afford it. Moreover, in our experience when a patient is critical, the family prefers to bring him/her home to die in a family setting and to avoid payment that is considered pointless.

We observed that 43 (28%) patients received a combination of ceftriaxone plus gentamicin, which is not recommended by any international guidelines and indicated an antibiotic misuse/overuse issue. Neither the Sudanese study nor the Kenyan one reported cases treated with this antibiotic scheme (20, 21), suggesting that this kind of overtreatment could be a result of the changing of the guidelines or a regional issue. The misuse of antimicrobial agents is a critical factor associated with AMR, which is a major threat to human health in sub-Saharan Africa, a high-burden region (22). In Jinka General Hospital, as in many other hospitals and health centers in Africa, there is no possibility of etiologic diagnosis by cultural tests. In a setting like that, empiric treatment is the only choice, so strict adherence to guidelines is mandatory to fight AMR (23). Many of the patients treated could also have viral pneumonia which is hard to distinguish from bacterial pneumonia (24). While the change in respiratory virus epidemiology during the COVID-19 pandemic is well-described in Western countries (25, 26), only a few are known in low-income countries where the lockdown policies were different (27). In Africa, the second wave of the COVID-19 pandemic was more severe than the first, and Ethiopia implemented public health and social measures in March 2020, which were subsequently reduced throughout the year (28).

In order to fight AMR in a high-burden region, we recommend scaling up policies to increase adherence to antibiotic first prescriptions even at hospital levels, such as posters in emergency departments with guideline summaries and frequent courses for general practitioners. Moreover, we suggest periodic monitoring of guideline adherence at the hospital level.

According to the guidelines, more than 80% of the patients presented at least one severe defining symptom. This is consistent with a Kenyan study that found an adherence rate of 57% on disease classification vs. 28% on treatment (21). We found a low proportion of severely malnourished patients (1%) because we only analyzed the admission to the Pediatric Ward and did not include patients admitted to the therapeutic feeding center (TFC) where most of the malnourished patients are managed. We chose not to include the TFC patients since they are strictly managed using the Ethiopian National Guidelines for Malnutrition (29) which contain detailed indications of how to use antibiotics in those patients. A large retrospective study in 14 hospitals in Kenya found that a weight-for-age Z-score less than−3SD and any grade of pallor was associated with death in children 2–59 months diagnosed with pneumonia, suggesting clinicians consider these risk factors in addition to the WHO criteria (30).

Malnutrition is a major problem in the Horn of Africa, with South Omo being one of the most affected areas (31, 32). While we are writing about the “perfect storm” of the COVID-19 pandemic, climate change and the wheat crisis are worsening the risk for this already extremely frail population (31, 33). A study conducted in South-West Ethiopia before and during the COVID-19 pandemic found a recovery rate from severe acute malnutrition (SAM) of 68% in children aged 6–59 months, which was lower than the minimum accepted international standard of 75%, and a lower recovery rate when SAM was comorbid with pneumonia (34). In this scenario, where the malnutrition epidemic is likely to increase pneumonia cases and antibiotic use, a quality assessment of their use is essential to containing AMR.

Some limitations have to be acknowledged. Since we performed a retrospective analysis, we could not evaluate the clinical assessment properly but only use the handwritten reports of symptoms on the patient's chart. Moreover, nearly one-third of the patients admitted with severe pneumonia were not assessed because of missing charts. Research in low-income countries is made complex by the lack of resources to ensure data collection, which could be improved by the development of regional surveillance systems (35, 36). Anyway, this selection was random, and we believe it has not affected our results. Since this is a monocentric study, it is not generalizable to other Ethiopian hospitals, but other previous studies showed alarming results. The strengths of our research are the access to data from a remote area too often forgotten by national and international policies but in desperate need of health improvement, methodological approach, and relatively large dataset.

## 5. Conclusion

Less than half of children received a treatment adherent to the WHO guidelines. Alternative antibiotic schemes did not prove superior to guideline-recommended schemes. Efforts should focus on understanding the causes and filling the gap between theory and practice. The fight against AMR should include a step up in quality monitoring systems in low-income countries where data are missing. However, none of the children admitted for pneumonia died.

## Data availability statement

The raw data supporting the conclusions of this article will be made available by the authors, without undue reservation.

## Ethics statement

The studies involving human participants were reviewed and approved by the Jinka University Ethical Committee. Written informed consent from the participants' legal guardian/next of kin was not required to participate in this study in accordance with the national legislation and the institutional requirements.

## Author contributions

AL, EH, and LP conceptualized and designed the study. AL, EH, LP, BT, and DM designed the data collection instruments and collected data. AL and CA were responsible for the analysis and interpretation of data. AL drafted the manuscript. CA and KT performed a critical revision of the manuscript and gave a significant contribution in their field of expertise. All authors approved the final manuscript as submitted and agree to be accountable for all aspects of the work.
